# Particle Size Dynamics: Toward a Better Understanding of Electronic Cigarette Aerosol Interactions With the Respiratory System

**DOI:** 10.3389/fphys.2018.00853

**Published:** 2018-07-09

**Authors:** Tomasz R. Sosnowski, Marcin Odziomek

**Affiliations:** Faculty of Chemical and Process Engineering, Warsaw University of Technology, Warsaw, Poland

**Keywords:** electronic cigarette aerosol, inhalation, deposition, hygroscopic growth, particle size distribution

## Abstract

The knowledge of possible acute and long-term health effects of aerosols inhaled from electronic cigarettes (ECs) is still limited partially due to incomplete awareness of physical phenomena related to EC-aerosol dynamics. This short review discusses the basic processes of aerosol transformation (dynamics) upon inhalation, indicating also the need for the accurate determination of the size of droplets in the inhaled EC-mist. The significance of differences in the aerosol particle size distribution for the prediction of regional deposition of inhaled mist in the respiratory system is highlighted as a decisive factor in the interactions of inhaled EC-aerosols with the organism.

## Introduction: the basic characteristics of EC aerosol

Electronic cigarettes (ECs), also known as electronic nicotine delivery systems (ENDS), have become popular consumer products (Palazzolo, 2013; Rom et al., 2015) being claimed both safer than tobacco cigarettes (TCs) and helpful in smoking cessation (Farsalinos and Polosa, 2014; McRobbie et al., 2014). However, there is still a debate about the acute and long-term health effects from inhalation of aerosol released by ECs (Vardavas et al., 2012; Schober et al., 2014; Farsalinos and Gillman, 2018). These questions arise also from incomplete knowledge of aerosol properties and dynamics after leaving the EC and entering the respiratory tract.

Aerosols emitted from ECs have special properties which should be taken into account during analysis of their dynamics and deposition in the respiratory system. Emitted (inhaled) aerosol is highly concentrated and contains mainly submicrometer-size particles. EC-aerosol, usually termed “vapor,” is composed of droplets of e-liquids, which contain mainly propylene glycol (i.e., 1,2-propanediol, PG), glycerol (i.e., propane-1,2,3-triol), nicotine, water, flavorings (if added to e-liquid), preservatives and also small amounts of by-products of thermal decomposition of some of these constituents (Goniewicz et al., 2014; Jensen et al., 2015). These droplets are surrounded by air and a mixture of vapors. The major e-liquid components have a high boiling point (PG: 180°C and glycerol: 300°C), hence a low volatility. The equilibrium saturated vapor pressure of PG at room temperature is below 17 Pa (0.13 mmHg) and of glycerol even less: 0.13 Pa (0.001 mmHg). Accordingly, the concentration of these vapors around droplets is low as compared to typical concentrations of water vapor which is characterized by the equilibrium pressure of ~2,350 Pa (17.6 mmHg; Maloney, 2008). Both PG and glycerol are hygroscopic which means that droplets can grow by taking-up the water vapor from the humid air.

Many experimental studies related to EC-aerosols try to adapt directly the methodology developed during decades of the research of the smoke emitted from TCs, often neglecting the discrepancies between both types of emissions. This short review is aimed to indicate similarities and differences in aerosols generated by ECs and TCs, and simultaneously to underscore the significance of particle size dynamics as the influential factor in the fate of inhaled aerosols inside the respiratory system. After analyzing basic thermodynamic and mass transfer effects in the inhaled EC-aerosols, the necessity of a correct size determination of particles released from electronic cigarettes will be highlighted.

## TCs vs. ECs—aerosol deposition and health effects

It is well-known that deposition of inhaled tobacco cigarette (TC) aerosols in the lungs has many undesirable health consequences. TC particles carry organics (VOCs) which are highly toxic and often carcinogenic. “Hot-spots” of smoke particle deposition are localized in the bronchial bifurcations (carinal regions) and are recognized as common places of lung cancer development (Balashazy et al., 2003). In contrast to TC, the vapor and droplets released from ECs are much less toxic which does not mean that they are completely safe for health (e.g., Kaisara et al., 2016; Lødrup Carlsen et al., 2018). The knowledge of their physical properties and behavior inside the body is incomplete and requires more studies for reasonable predictions of preferred sites of their deposition in the respiratory system. Regional doses of deposited aerosols inhaled from TCs and ECs have been compared e.g., by Manigrasso et al. (2015) and Pichelstorfer et al. (2016) who found from numerical computations that numbers of EC droplets deposited both in pulmonary and tracheobronchial regions were approximately two-fold higher than the numbers of deposited TCs particles in these regions. The authors claim that slight differences in puffing topography between TCs and EC are without effect on the regional deposition, however other phenomena such as droplet coagulation and hygroscopic growth in EC aerosol have the most prominent influence on enhanced regional deposition comparing to TCs particles. Interestingly, according to Pichelstorfer et al. (2016) in both types of cigarettes nicotine is primarily absorbed from gaseous/vapor phase, not from deposited particles or droplets. Sosnowski and Kramek-Romanowska (2016) calculated the influence of breathing parameters on the regional deposition of EC aerosol (CMD ~200 nm) using Multiple-Path Particle Dosimetry model and they found that deeper and slower inhalation with a breath-hold enhances droplet deposition in the pulmonary region, probably due to stronger diffusive effect. Hygroscopic growth effects were neglected in these computations. These authors also tested the influence of mean droplet size on the regional deposition of EC aerosols and they found that increased size at the inlet enhances deposition mainly in the head airways while the deposition in bronchial and pulmonary regions remains practically unchanged. According to Manigrasso et al. (2015), maximum EC aerosol deposition is predicted in generations no. 16–23 of the stochastic lung model, i.e., in the small airways including alveoli. Such estimations are similar to the ones obtained for TC smoke from computations on Weibel lung model (Robinson and Yu, 2001). In spite of similar deposition pattern and “hot-spots” of deposition in the bronchial bifurcation region (Balashazy et al., 2003), the EC droplets are expected to be much less toxic since they do not contain mutagenic compounds originated from combustion. Accordingly, the risk of getting lung cancer with EC use was claimed to be significantly reduced both for active and passive vaping (Scungioa et al., 2018).

In spite of that, localized deposition of inhaled EC droplets and absorption of vapor phase has certain physiological consequences. Both nicotine delivery rate and local side effects caused by interactions of inhaled compounds with the mucus and lung surfactant must be taken into consideration together with the direct influence of inhaled compounds on the epithelial cells. These issues have been treated by several review papers (Bengalli et al., 2017; Palazzolo et al., 2017; Shields et al., 2017; Glantz and Bareham, 2018).

## The role of inhalation pattern

**Figure 1:**
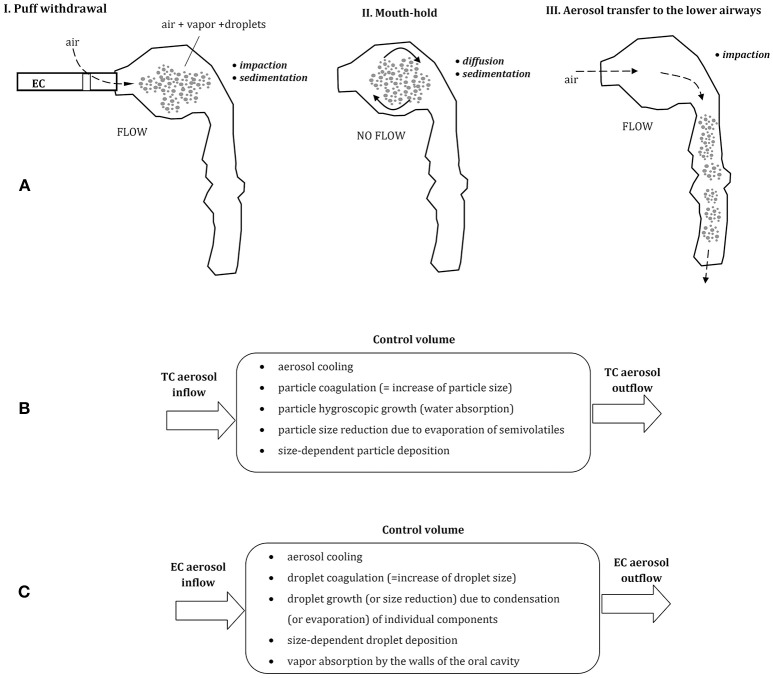
**(A)** Three phases of vaping: puff withdrawal, aerosol mouth-hold, and aerosol inhalation (the predominant aerosol droplets deposition mechanisms are indicated). Below—the comparison of thermodynamic and mass transfer effects after inhalation of aerosol from tobacco cigarette, TC **(B)** and electronic cigarette, EC **(C)**; all these processes take place simultaneously inside a control volume (e.g., a small segment of the oral cavity).

Since ECs are most often used by previous or current smokers, the manner of aerosol inhalation remains quite similar (habitual) as in smoking, although some discrepancies have been also reported (Behar et al., 2015). Typically, in both types of cigarettes, the aerosol (formed by TC smoke or EC “vapor”) is initially introduced to the mouth as a “puff,” and then—after a few-second mouth-hold—it is inhaled to the lungs, (Figure 1A). Accordingly, these two periods of: (i) drawing a puff and (ii) the mouth-hold, provide a certain time for a change of initial aerosol properties due to thermodynamic and mass transfer effects. In should be noted that such manner of aerosol inhalation is substantially different than tidal breathing or inspiratory patterns typically analyzed in the inhalation issues of occupational safety or inhalation therapy. It is also a reason why quantitative models which relatively well can predict lung deposition of aerosols in both mentioned areas, are hardly applicable to ECs without substantial modifications (Sosnowski and Kramek-Romanowska, 2016; Asgharian et al., 2018).

In spite of comparable inhalation pattern using TCs and ECs, aerosol dynamics in the respiratory system is different due to dissimilar properties of each aerosol. Smoke produced by combustion of tobacco in TCs is composed of fine solid and semi-volatile particles suspended in air, while ECs produce a mist of liquid droplets suspended in the mixture of vapors and air. EC-droplets are formed by condensation of a vapor produced by heating of e-liquid, and they contain different proportions of e-fluid constituents and by-products. This dissimilarity between inhaled aerosols has important consequences for the dynamics of inhaled particles in the respiratory system, which will be discussed below. In addition, as demonstrated by Trtchounian et al. (2010) and supported by Sosnowski and Kramek-Romanowska (2016), TCs and ECs have different internal resistance to the airflow, and smoking is easier (i.e., requires less respiratory effort) than vaping. This observation has further consequences, since a higher limitation of airflow during inhalation leads to a constriction of the oral cavity, i.e., to the reduction of its volume. It was shown by Ehtezazi et al. (2004), based on CT scans of the upper respiratory tract geometry during air inspiration via inhalers with different internal aerodynamic resistance. Higher airflow obstruction also chokes the flow, so the mean velocity of inhaled aerosols inside the oral cavity is lower and aerosol residence time in this region is longer. Both effects (a change in oral geometry and a reduced airflow) influence aerosol particles dynamics and deposition inside the oral cavity and beyond.

## Aerosol dynamics after inhalation

Puff volume from ECs is highly variable and can be between ~30 and more than 350 mL depending on vaper (Robinson et al., 2015). Also, the flow rate during puffing and the puffing time is scattered (~25–100 mL/s and 0.7–6.9 s, respectively). It is generally agreed that aerosol particles inhaled from tobacco cigarettes (TCs) and droplets inhaled from ECs have a similar size distribution and are of similar or slightly different concentration (Ingebrethsen et al., 2012; Pellegrino et al., 2012; Fuoco et al., 2014; Glasser et al., 2017). Undoubtedly, they differ in chemical composition and thermodynamic state. Aerosol dynamics after puffing can be schematically depicted for both types of cigarettes in Figures 1B,C. In case of EC-aerosol, droplets remaining for some period in the oral cavity can evaporate (which decreases their size), the surrounding vapor may condense on droplets' surface and droplets may coagulate (both processes increase the average droplet size). Simultaneously, the vapor may be absorbed by the walls of the oral cavity which reduces the vapor partial‘ pressure (i.e., concentration) in the gas phase, hence changes the driving force for evaporation/condensation processes. These thermodynamic phenomena are accompanied by mass transfer effects i.e., particle displacement and deposition on the surface of the oral cavity. It is clear, that properties and concentration of inhaled aerosol are dynamically altered during a relatively short period of aerosol residence in the upper airways, and this effect has also a strong impact on droplets distribution and deposition after aerosol transfer to the lower airways.

Aerosol dynamics in the upper airways was recently described mathematically by Asgharian et al. (2018). This approach accounts for the effects of multi-component evaporation/condensation of e-liquid components in addition to droplet coagulation (coalescence) and simultaneous deposition of droplets and vapors during puff withdrawal and mouth-hold of inhaled EC-aerosol.

The change of mass *(m)* of a single droplet during the period when it changes the position *(z)* is a sum of coagulation *(CO)* and evaporation/condensation *(EVC)* effects, i.e.,

(1) $$\begin{array}{r} \frac{dm}{dz}=\;\frac{dm}{dz}\;{|}_{CO}+\;\frac{dm}{dz}\;{|}_{EVC} \end{array}$$

The mass change due to coagulation $\left(\;\frac{dm}{dz}\;{|}_{CO}\right)\;$ can be found by solving the transport equation:

(2) $$\begin{array}{r} \frac{\partial c}{\partial t}+u\frac{\partial c}{\partial z}=-\beta c^{2} \end{array}$$

where *c* denotes the concentration of droplets with a given size, *u*—the mean aerosol (droplet) velocity in the control volume, *t—* the time, and β is the coagulation kernel, which can be calculated based on the air and particle properties.

Effects associated with the evaporation/condensation *(EVC)* depend on droplet size (curvature) and composition. For very small droplets there is an increase of partial pressure of vapor above the droplet surface (so-called, Kelvin effect) which accelerates the evaporation rate (Ho, 1997):

(3) $$\begin{array}{r} P_{b}=P_{s}\left(T\right)\exp\left(\frac{2\sigma }{\rho R_{M}Tr}\right) \end{array}$$

where:

*P*_*b*_—the equilibrium vapor pressure above the curved surface of a droplet, *P*_*s*_—the equilibrium vapor pressure above a flat liquid surface, ρ, and σ—the density and surface tension of the liquid, respectively and *R*_*M*_—the individual gas constant (i.e., universal gas constant divided by the molar mass). Numerical results obtained from Equation (3) for individual EC constituents show that an increase of the equilibrium vapor pressure due to surface curvature becomes essential (*P*_*b*_*/P*_*s*_>>1) only for very small droplets (*r* < 5 nm), and the effect is more important for glycerol than for PG, while it is the smallest for water. It is therefore plausible that for the majority of droplets in EC-aerosol, the evaporation is not accelerated by Kelvin effect.

According to Asgharian et al. (2018), for each volatile chemical component *i* of a droplet, the combined evaporation/condensation effect can be described as:

(4) $$\begin{array}{rcl} \frac{dm_{i}}{dz} & = & \frac{AD_{i}c_{i,\;max}}{Q}\text{Sh }{\left(\frac{6\pi^{2}m}{\rho }\right)}^{\frac{1}{3}}\frac{\text{Kn}+1}{1.3325{\text{Kn}}^{2}+1.71\text{Kn}+1}\\ \times \left[S_{i}-\frac{T_{\infty }}{T}a_{i}x_{i}\text{exp}\left(B_{i}\right)\right] \end{array}$$

where, for each component *i* of the liquid or vapor mixture, *D*_*i*_ is diffusion coefficient in air, *L*_*i*_—the latent heat of evaporation, *S*_*i*_—the saturation ratio, *M*_*i*_—the molecular weight, and *a*_*i*_—the activity coefficient. *T*_∞_ is the temperature of the surrounding gas, *R*—the universal gas constant. Kn denotes the Knudsen number for a droplet with the given size, and Sh is the Sherwood number (it equals 2 if the convective mass transfer can be neglected). Parameter *B*_*i*_ in Equation (4) is expressed as:

(5) $$\begin{array}{r} B_{i}=\frac{4\sigma_{i}M_{i}}{\rho_{i}RT}{\left(\frac{\pi \rho }{6m}\right)}^{\frac{1}{3}}+\frac{L_{i}M_{i}}{R}\left(\frac{1}{T_{\infty }}-\frac{1}{T}\right) \end{array}$$

The total change in droplet mass is obtained by summing the results for all constituents of liquid droplets and the vapor:

(6) $$\begin{array}{r} \;\frac{dm}{dz}\;{|}_{EVC}=\underset{i}{\sum }\frac{dm_{i}}{dz} \end{array}$$

Due to the heat exchange between aerosol (droplets) with temperature *T* and the surrounding environment *(T*_∞_*)*, the energy equation has to be simultaneously solved. Numerical solution of the model presented above provides temporal (or spatial) evolution of droplet size, composition, concentration, and temperature. According to the results presented by Asgharian et al. (2018), the EC-vapor inhaled at 87°C is cooled to the body temperature during a short time of puff withdrawal when the aerosol penetrates initial 10 cm inside the oral cavity. The model also predicts that the uptake of PG, glycerin and nicotine vapors by the walls of the oral cavity noticeably enhances the evaporation from droplets due to the removal of these components from the gas phase (i.e., increasing the driving force for the evaporation). Nevertheless, the hygroscopic growth of droplets due to absorption of water vapor predominates, so the net effect described by the LHS Equation (1) is positive. Accordingly, a droplet with the initial size of, e.g., 500 nm is expected to grow to almost 900 nm. Calculation results also suggest that the total uptake of EC-droplets and EC-vapor in the oral cavity during combined phases of puff withdrawal and aerosol mouth-hold is around 5%, while the highest fractional collection is observed for PG (~6%), nicotine (4.5%), and glycerin (4%). As a result, roughly 95% of inhaled EC constituents of inhaled vapor become available for the transfer to the lower airways. Numerical data presented by Asgharian et al. (2018) confirm the growth of 0.5 μm EC-aerosol droplet in the mouth, however, these authors do not discuss the influence of droplet initial size and composition on this phenomenon. Impact of the initial particle size may be high as previously demonstrated for TC-aerosols (Asgharian et al., 2014). In case of TC-smoke, some particles can partly evaporate (semi-volatiles, Figure 1B), so particle growth usually predominates. The process has some analogy to the one discussed for EC-aerosol dynamics. TC-aerosol particles grow inside the oral cavity with the rate which is dependent on their initial size. Numerical predictions show that after 1 s period of remaining in this volume, particles with 0.1 μm initial diameter slightly increase their size, however, 0.5 μm particles become larger by 50%, while 1 μm particles grow almost two-fold. This process is driven mainly by the absorption of condensing water vapor in a humid environment of the oral cavity.

In general, inhaled aerosol particles or droplets are deposited in the respiratory system mainly due to the mechanisms of gravitational settling (sedimentation), diffusion and impaction, depending on particle size and local flow velocity (e.g., Pirozynski and Sosnowski, 2016; Sosnowski, 2016). It should be noted then that an increase of particles size reduces their deposition due to Brownian diffusion but accelerates gravitational settling and inertial deposition during aerosol flow (Figures 1A,C). As a consequence, a some inhaled aerosol particles are always deposited in the oral cavity, however, this fraction is dependent on the initial size of inhaled aerosol particles.

Taking into account discussed-above heat and mass transfer effects it becomes clear that the initial particle size distribution of inhaled EC-aerosol is a key factor in the correct prediction of aerosol dynamics which, in turn, is required for the prognosis of regional particle deposition and absorption of vaporized components. This finding underscores the problem of the appropriate size determination of droplets released from ECs.

## Particle size measurement techniques and their applicability to EC mist

The unique properties of aerosol released from ECs require proper methods of particle size analysis. Literature data clearly show that the determined particle size depends on the applied measuring equipment. Since particles/droplets in both TCs and ECs are formed by nucleation (i.e., combustion in TCs and vapor condensation in ECs), their primary size may be in the nanometer scale. Meanwhile, the concentration of freshly formed aerosol is very high which should favor nanoparticle coagulation just after nucleation.

The most common measuring technique of aerosol nanoparticles is based on their size-dependent mobility in the electrostatic field. A device known as DMA (differential mobility analyzer) is usually embedded within the larger systems known as SMPS (scanning mobility particle sizer) of DMPS (differential mobility particle sizer). During the DMA measurement nanoparticles with a given diameter range can be extracted from the aerosol stream by applying a certain voltage which deflects their path and allows drawing them to the particle counter. Next, each nanoparticle becomes a nucleus of condensation of an organic solvent (e.g., butanol), and grows to the size which can be detected optically (in CPC—condensation particle counter). By scanning many predefined voltage values, nanoparticles with different sizes can be sampled and counted separately, so finally, the information on the aerosol particle size distribution is derived. Typically the mentioned devices are capable to determine particles in the size range of 10–1,000 nm (Konstantinos et al., 2017). This methodology has several limitations in the respect to EC aerosols:

1. The residence time of droplets in the device is long enough to allow the droplets to change their size during the measurement by already mentioned thermodynamic mechanisms.
2. The aerosol is diluted inside DMA by the additional stream of sheath air. This undoubtedly influences droplets evaporation and coagulation rate comparing to the real situation in the released/inhaled EC-aerosol cloud.
3. The prolonged scanning of different voltages is justified for continuous and stable aerosol sources. ECs release aerosol for a short period of time (a puff), so finding the complete aerosol size distribution requires the measurements on many individual puffs—this raises a question of stability and reproducibility of this aerosol source.
4. The results are time-averaged, so they do not allow track the dynamics of puff release.

Ingebrethsen et al. (2012) determined by such system that the size of EC-droplets is in the range of 10–50 nm. At the same time, it was found that the total mass of droplets calculated according to the measured sizes was orders of magnitude lower than the mass determined by the gravimetric method. This confirms the problem of aerosol dilution in case of ECs. According to the different, supplementary method—the spectral transmission, which does not require aerosol dilution—the size of the same droplets was in the range of 210–380 nm (Ingebrethsen et al., 2012). Similar size range was also found with other techniques which are discussed below (Alderman et al., 2014; Sosnowski and Kramek-Romanowska, 2016; Sundahl et al., 2017).

Impactors and impingers are aerosol classifiers operated on inertial principle, which reflects differences in particles resistance to the change of airflow direction. Larger particles with high inertia are separated from the air stream by impaction with the collection surface (solid or liquid) while smaller ones are transported with air to the further impaction stages (Mitchell and Nagel, 2004). The standard devices of this type can classify particles in the size range of 0.1–15 μm (their collected mass is typically determined by the selective instrumental methods, e.g., HPLC). Smaller particles/droplets may be separated in the impactors or impingers by applying high airflows which means a dilution of tested aerosol and a higher pressure drop in the device. Both effects can result in measurement errors. Another choice for nanosize particles is a multi-stage impactor operated under reduced pressure. In the device known as electrostatic low-pressure impactor (ELPI), the amount of collected particles is determined by measuring their total electric charge. In the modern ELPI system the measuring range is wide (6 nm−10 μm), however for EC-aerosols the low pressure (down to 40 mbar) under which the device is operated may accelerate droplet evaporation during the measurement, resulting in the underestimation of the measured droplet diameter (Jarvinen et al., 2014; Konstantinos et al., 2017). Other limitations of impactors in EC aerosols determination are that (i) they provide only time-averaged data and (ii) the particle size assessment is resource-, labor-, and time- consuming.

A number of measuring devices utilize optical systems (aerosol spectrometers) with different operation principles. Spectrometers provide the real-time particle size determination, so they may be considered applicable to ECs (and TCs) aerosols, although they usually cannot detect particles smaller than 100–200 nm. Time-of-flight analyzers measure the time needed for aerodynamic particle motion between two laser beams. According to the measuring principle, these methods require a diluted aerosol to distinguish individual particles. The same problem is related to laser scattering methods which are based on the detection of optical signal from a single particle at a time. Therefore, in spite of a low e-liquid volatility and the average size of EC- aerosol droplets not favoring the Kelvin effect, size measured by such systems may be underestimated due to the evaporation losses (Alderman et al., 2014).

In view of that, the best measuring instruments for EC-aerosols should have low internal resistance and require no additional dilution with air. Laser diffraction spectrometers seem more suitable for studies of concentrated aerosols such as those released by TCs or ECs (Sosnowski and Kramek-Romanowska, 2016) Particle size distribution is determined here, after analysis of interference pattern produced by a whole aerosol cloud which must sufficiently obscure the laser light. Application of Mie or Fraunhofer theory allows determining the contribution of particles with different size (de Boer et al., 2002). Moreover, due to a dense matrix of light detectors, the quasi-continuous distribution data in a broad particle range size can be obtained. The signal sampling rate can be very high (up to kHz) which allows to test short-lasting particle clouds and trace aerosol dynamics. The only limitation of the measurement is the necessity of the exact knowledge of the refractive indexes of measured particles and the continuous phase.

Results obtained for TCs and ECs by various methods of aerosol size determination, also during application of variable experimental condition are listed in Table 1. These data indicate that EC droplets measured with DMPS or FMPS systems have the count median diameter (CMD) typically in the range of 100–200 nm, which is slightly changing with the dilution. This range also corresponds to TC aerosols when they are determined with the same methodology. Results from other measuring devices, i.e., optical counters, impactors, diffraction, and spectral transmission spectrometers, show higher values of CMD (180–400 nm). Interestingly, data for the equilibrated EC aerosol measured with SMPS by Zhang et al. (2013) are similar to the results obtained with other devices. This confirms that typical SMPS/FMPS data may underestimate EC droplet size due to additional dilution with a sheath flow.

**Table 1:** Reported particle size emitted from tobacco and electronic cigarettes obtained with different measuring techniques and conditions (CMD, count median diameter; MMAD, mass median aerodynamic diameter).

**Method of aerosol generation/measurement technique**	**Particle/droplet size**		**Literature**
	**Electronic cigarettes**	**Tobacco cigarettes**	
Puffing Machine/Spectral Transmission Method (non-diluting conditions)	CMD = 210–380 nm		Ingebrethsen et al., 2012
Puffing Machine/Differential Mobility Spectrometer—DMS500 (electrical mobility analysis—high dilution ratio)	CMD = 10–50 nm		Ingebrethsen et al., 2012
Puffing Machine/Differential Mobility Spectrometer—DMS500 (electrical mobility analysis—high dilution ratio)		CMD = 145–189 nm	Ingebrethsen and Alderman, 2011
Puffing Machine/Scanning Mobility Particle Sizer (SMPS TSI3936)	CMD = 120–180 nm (single puff; droplets counted immediately after leaving e-cigarettes) CMD = 400 nm (steady-state; aerosol suspended in a chamber)	CMD = 100–600 nm	Zhang et al., 2013
Constant air flow rate (2 L/min)/MOUDI cascade impactor (non-diluting conditions)	CMD = 260–320 nm		Alderman et al., 2014
Constant air flow rate (1.08 L/min)/Next Generation Impactor	MMAD = 500–900 nm		Sundahl et al., 2017
Constant air flow rate (5 L/min) /Diffraction Spectrometer (non-diluting conditions)	CMD = 180–220 nm		Sosnowski and Kramek-Romanowska, 2016
Constant air flow rate/Fast Mobility Particle Sizer (FMPS TSI3091) (electrical mobility analysis—high dilution ratio)	CMD = 107–143 nm	CMD = 165 nm	Marini et al., 2014
Volunteering smokers/Optical Particle Counter and Portable Aerosol Mobility Spectrometer	CMD = 191 ± 41 nm (low dilution ratio) CMD = 45 ± 12 nm (high dilution ratio)		Meng et al., 2017
Volunteering smokers, aerosol suspended in an emission test chamber/Fast Mobility Particle Sizer (FMPS TSI3091) (electrical mobility analysis—high dilution ratio)	Size distribution peak at 60 nm	Size distribution peak at 100 nm	Schripp et al., 2013

Another important issue in aerosol particle size analysis is the appreciation of the difference between number- and volume-based particle size distributions. Some measuring systems provide data derived from particles counting (e.g., DMA+CPC) while others are based on their volumetric contribution. Since the particle volume is proportional to *r*^3^, it is obvious that the mean (or median) particle size evaluated regarding the volumetric contribution will be always higher than the mean (or median) diameter/radius determined using particle counts. The relationship between mass median diameter (MMD) and count median diameter (CMD) for different particle size distribution has been recently explained by Pirozynski and Sosnowski (2016). For instance, as shown in studies by Sosnowski and Kramek-Romanowska (2016), the median volumetric diameter of tested EC-aerosol was close to 400 nm, while the recalculated median number diameter was <200 nm. The difference in this values is essential if one uses them as entry data in the modeling of EC-aerosol dynamics in the respiratory system (see section The Role of Inhalation Pattern). It may be also noted that if the mass of inhaled aerosol is concerned, nanoparticles/nanodroplets can be neglected as their mass contribution (even if they are at prevalence in number) is relatively low comparing to micrometer-sized particles. On the other hand, the mass may be not good metrics of particle influence on the respiratory system if local effects on the lung surface are considered (Sosnowski, 2018).

## Conclusions

Possible health outcome and nicotine delivery from ECs depend on physical properties of the emitted particles and vapors. This short review highlighted the problem of the assessment of EC-aerosol dynamics in relation to the further fate of inhaled aerosol in the respiratory system, i.e., regional droplet deposition and vapor absorption. Even though inhalation of EC aerosols is believed to be safer for health than smoking, it is important to understand the distribution of particle deposition in the human respiratory system. Due to the possibility of aerosol transformation (droplet evaporation, coagulation, and growth) immediately after emission from EC, the need for correct droplet size determination becomes essential. A more thorough understanding of particle size dynamics after aerosol release and during inhalation should improve the debate on any possible health effects of inhaled EC-aerosols.

## Author contributions

TS prepared the overall conception of the paper, and took part in: (i) literature review, (ii) analysis of aerosol dynamics and fate after inhalation, (iii) preparation of the manuscript, (iv) preparation of the drawings. MO took part in: (i) literature review, (ii) analysis and description of measuring techniques used for EC mist characterization, (iii) preparation of the manuscript.

### Conflict of interest statement

During preparation of this paper MO was involved in EC-aerosol research funded by NCN project 2015/19/D/ST8/00822. The remaining author declares that the research was conducted in the absence of any commercial or financial relationships that could be construed as a potential conflict of interest.
